# Fatal Human Infection with Evidence of Intrahost Variation of Eastern Equine Encephalitis Virus, Alabama, USA, 2019

**DOI:** 10.3201/eid2707.210315

**Published:** 2021-07

**Authors:** Holly R. Hughes, Jason O. Velez, Emily H. Davis, Janeen Laven, Carolyn V. Gould, Amanda J. Panella, Amy J. Lambert, J. Erin Staples, Aaron C. Brault

**Affiliations:** Centers for Disease Control and Prevention, Fort Collins, Colorado, USA

**Keywords:** Eastern equine encephalitis virus, immunosuppression, rituximab, quasispecies, SNV, intrahost variation, viruses, Alabama, USA, vector-borne infections, zoonoses

## Abstract

Eastern equine encephalitis virus (EEEV) is an arbovirus in the family *Togaviridae*, genus *Alphavirus*, found in North America and associated with freshwater/hardwood swamps in the Atlantic, Gulf Coast, and Great Lakes regions. EEEV disease in humans is rare but causes substantial illness and death. To investigate the molecular epidemiology and microevolution of EEEV from a fatal case in Alabama, USA, in 2019, we used next-generation sequencing of serum and cerebrospinal fluid (CSF). Phylogenetic inference indicated that the infecting strain may be closely related to isolates from Florida detected during 2010–2014, suggesting potential seeding from Florida. EEEV detected in serum displayed a higher degree of variability with more single-nucleotide variants than that detected in the CSF. These data refine our knowledge of EEEV molecular epidemiologic dynamics in the Gulf Coast region and demonstrate potential quasispecies bottlenecking within the central nervous system of a human host.

In North America, eastern equine encephalitis virus (EEEV) causes disease in equids, domestic birds, and humans (*1*,*2*). The virus is maintained in an enzootic cycle between passerine avian amplification hosts and *Culiseta melanura* mosquitoes as the principal mosquito vectors (*3*). EEEV infections in humans and equids result from spillover from the enzootic transmission cycle or by the bites of bridge vectors that can become infected during epizootics. In humans and equids, viremia does not develop at sufficient levels to infect additional mosquito vectors; however, the disease can be severe because of the neurotropic nature of the virus (*4*).

In the New England region, cases of eastern equine encephalitis (EEE) resulting from EEEV infection in humans are seasonal and are typically reported during July–October (*5*); in Florida, EEEV transmission persists all year (*6*). The first case of EEE in a human was identified in Massachusetts in 1938 after an epizootic among horses (*2*). Before 2019, the last major EEE epidemic occurred in New Jersey in 1959; a total of 32 cases in humans were reported (*7*). During 2003–2018, an average of 8 (range 4–21) EEE cases/year in humans were reported to the Centers for Disease Control and Prevention (CDC) (*8*). Although reports of EEE in humans are rare and the proportion of inapparent infections is high (*7*), the case-fatality rate for patients with reported cases of neuroinvasive EEE is estimated to be 30% (*9*) and the rate of long-term sequalae in survivors is high, making EEEV infections a substantial public health concern. In 2019, an unprecedented epidemic of EEE across the eastern and upper midwestern United States resulted in 38 confirmed cases in humans, most in Massachusetts and Michigan (*8*).

EEEV is highly genetically conserved; a single major lineage has been circulating since 1933 (*10*). Phylogenetic studies have shown substantial genetic diversity among isolates of Madariaga virus, the virus most closely related to EEEV (*11*). A recent study demonstrated more EEEV genetic diversity among strains in Florida, most likely resulting from year-round transmission and more geographic mixing of EEEV than what is seen in northern states (*12*). 

We investigated the molecular epidemiology of EEEV sequences from 1 patient infected with EEEV in Alabama, an area with historically limited genetic information about EEEV. In addition, we evaluated intrahost virus diversity of EEEV in the patient and report genetic diversity of virus in the blood compared with the central nervous system (CNS). All methods followed manufacturer’s recommended protocols unless otherwise noted.

## Methods

### The Patient

The patient was a woman in her 60s who had lymphoma, for which she was receiving rituximab. She was active and working outdoors until September 2019, when she experienced lethargy and malaise. Approximately 1 week after symptom onset, she was found at home unresponsive and was transferred to the hospital. Her evaluation at the hospital indicated suspected viral encephalitis, but test results for numerous viral and bacterial etiologies, including testing of cerebrospinal fluid (CSF) by BioFire panel (BioFire Diagnostics, LLC, https://www.biofiredx.com), were negative; CNS lymphoma also was ruled out. The patient received broad-spectrum antimicrobial drugs and intravenous immunoglobulin, but her condition did not improve. She lapsed into a coma and never regained consciousness. Life support was discontinued, and she died 43 days after initial illness onset.

### Samples

We extracted RNA from 140 μL of serum and CSF from the patient by using the QIAamp Viral RNA Mini Kit (QIAGEN, https://www.qiagen.com). We performed real-time reverse transcription PCR (RT-PCR) to detect viral RNA from the endemic encephalitic arboviruses, West Nile virus (WNV), and EEEV. We performed EEEV real-time RT-PCR as previously described (*13*) by using 10 μL of RNA and a QuantiTect Probe RT-PCR Kit (QIAGEN).

### Library Preparation and Sequencing

We generated complementary DNA by using the Ovation RNA-Seq System V2 (NuGen, https://www.nugen.com). For whole-genome sequencing, we used the Ion Torrent Personal Genomic Machine system. We prepared libraries by using the Ion Plus Fragment Library Kit barcoded with the Ion Xpress Barcoding Kit and quantified by using the Ion Library TaqMan Quantitation Kit (all by Thermo Fisher Scientific, https://www.thermofisher.com). We prepared sequencing templates by using the Hi-Q View OT2 kit with the Ion One Touch 2 system (both by Thermo Fisher Scientific) and completed sequencing by using a Hi-Q View Sequencing Kit (Thermo Fisher Scientific). We loaded templated ion sphere libraries onto 318 Chips V2 and sequenced them by using the Ion Torrent PGM system (both by Thermo Fisher Scientific). We deposited virus sequences from this study into GenBank (accession nos. MT782294 and MT782295).

### Whole-Genome Analysis

We loaded Fastq files (quality phred Q>20) into the CLC genomic workbench version 12 (QIAGEN) and assembled genomes by using de novo assembly. We identified viral contigs by using BLAST (https://blast.ncbi.nlm.nih.gov/Blast.cgi) and completed alignments by using the de novo assembled consensus sequences (GenBank accession nos. MT782294 and MT782295) and Bowtie2 version 2.3.4.1 (https://github.com/BenLangmead/bowtie2) with paired-end, sensitive local parameters. We removed PCR duplicates with MarkDuplicates (Picard Tools; Broad Institute, https://broadinstitute.github.io/picard). We calculated mutational frequency by using custom R scripts over possible nucleotide variables (A, U, C, G, –) according to the method described by Matsushita et al. (*14*) and called variants by using default settings of the software LoFreq (v2.0) requiring 2% frequency with a minimum of 100 reads (*15*).

We inferred phylogenies by using MEGA v7 (*16*). We downloaded reference EEEV complete genomes from GenBank (December 4, 2019) and codon aligned complete coding sequences by using ClustalW (*16*). 

 We completed phylogenetic inference by using a maximum-likelihood algorithm with 1,000 bootstrap replicates and the general time-reversible model with gamma distributed rate variation and invariable sites, as determined by the model fit test in MEGA (https://www.megasoftware.net). We used Bayesian inference with BEAST (https://beast.community) and a Markov chain Monte Carlo approach of 100 million generations to confirm the maximum-likelihood tree topologies.

## Results

### Encephalitic Arboviruses in Clinical Samples

Serum and CSF specimens collected on day 24 of the patient’s illness were sent to the CDC Arboviral Diagnostic and Reference Laboratory (Division of Vector-Borne Diseases, National Center for Emerging and Zoonotic Infectious Diseases, Fort Collins, CO, USA), for further evaluation of potential arboviral etiologies. Serum was negative for IgM against La Crosse virus, Jamestown Canyon virus, Powassan virus, and EEEV; neutralizing antibodies against EEEV were not detected. Test results for WNV and Saint Louis encephalitis virus IgM performed at another laboratory were reportedly negative. CSF was negative for IgM against Powassan virus and EEEV. Because the patient was receiving rituximab therapy, which can suppress antibody production, real-time RT-PCR testing was performed and found to be negative for WNV RNA; however, EEEV RNA was detected in serum and CSF. Quantification cycle (Cq) values were 27.9 (serum) and 20.5 (CSF).

### Genomic and Phylogenetic Analyses

**Figure 1:**
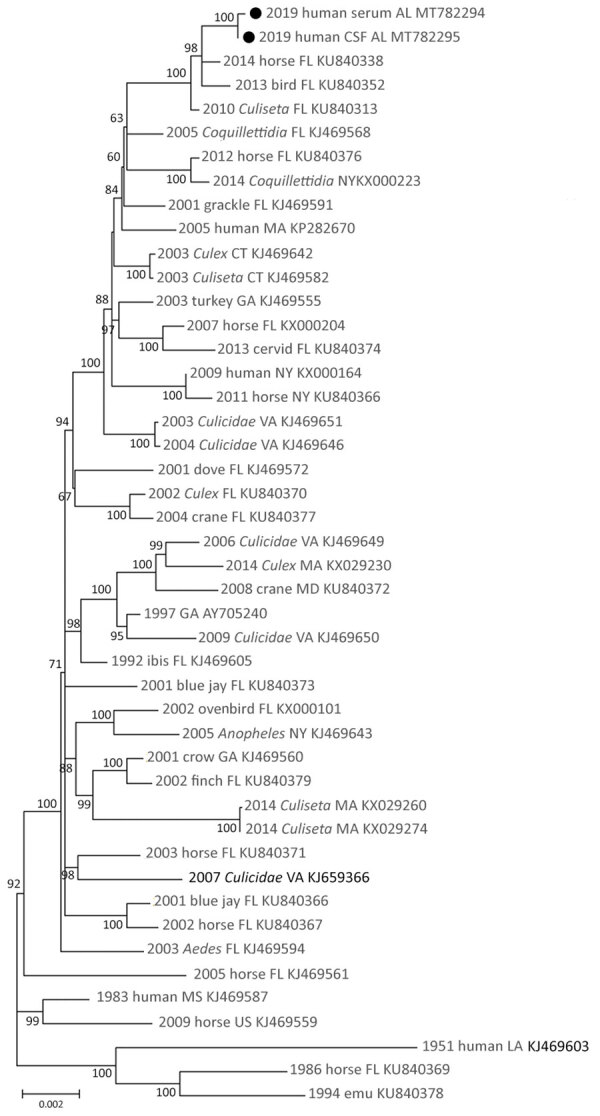
Maximum-likelihood phylogeny of eastern equine encephalitis virus from a woman in Alabama, USA, 2019 (solid circles), and reference sequences, based on complete coding region sequences. Nucleotide coding sequences of the full genome of eastern equine encephalitis viruses isolated during 1951–2019 were codon aligned and phylogenies inferred with general time reversible plus gamma plus proportion of invariable sites. Taxa are labeled with year of isolation, host, US state of isolation, and GenBank accession number. Branches are labeled with bootstrap support values as a percentage of 1,000 replicates. Branches with <50% bootstrap support are collapsed; only branches with >60% support are labeled. Branch lengths are drawn to scale and measured in the number of substitutions per site.

Complete genome sequences of EEEV were obtained from each specimen: serum (520× coverage) and CSF (2,689× coverage). The EEEV consensus sequence from the serum shared 99.79% nt identity with EEEV sequences from Florida isolated in 2010 (GenBank accession no. KU840313) and 2014 (GenBank accession no KU840338). The EEEV consensus sequence from the CSF shared 99.81% nt identity with these same reference sequences. Maximum-likelihood phylogenetic analysis supported these findings and placed the derived sequences from the serum and CSF in a well-supported clade with EEEV isolated from northern Florida in 2013 and 2014 (Figure 1). These data suggest that the virus sequences obtained in this study are similar to EEEV circulating in the southeastern United States since 2010.

### Intrahost Variability of EEEV in Serum and CSF

Intrahost variability was measured by detecting single-nucleotide variants (SNVs) in each specimen (Table). We detected 19 SNVs in the serum: 11 in the nonstructural genes, 7 in the structural genes, and 1 in the 3′ untranslated region (UTR). Of the 19 SNVs identified in the serum, 4 were synonymous. In contrast, 12 SNVs were identified in the CSF: 3 in the nonstructural genes, 5 in the structural genes, and 4 in the 3′ UTR. Of the 12 SNVs in the CSF, 1 was synonymous. When comparing the serum and CSF, we identified 4 SNVs in both specimens: 1 synonymous SNV at position 1322 in nonstructural protein 1 (NSP1), 2 nonsynonymous SNVs at positions 4443 in NSP3 and 9200 in envelope protein 2 (E2), and 1 SNV in the 3′ UTR at position 11312.

**Table:** Comparison of intrahost variability of eastern equine encephalitis virus variants in serum and cerebrospinal fluid from a woman in Alabama, USA, 2019*

Reference position†	Serum consensus/SNV	Serum SNV frequency, %	CSF consensus/SNV	CSF SNV frequency, %	Protein	Serum SNV amino acid substitution	CSF SNV aa substitution
646	T/C	4.42	T/–		NSP1	I208T	
775	C/T	14.40	T/–		NSP1	T251I	
778	T/A	9.13	T/–		NSP1	L252Q	
1322	T/C	12.18	T/C	6.52	NSP1	P433	P433
1326	A/C	5.46	A/–		NSP1	T435P	
2719	T/–		T/C	3.23	NSP2		L366P
2866	G/A	5.59	G/–		NSP2	R415H	
2871	G/C	6.74	G/–		NSP2	E417Q	
4443	T/C	7.75	T/C	7.34	NSP3	W147R	W147R
4445	G/A	14.05	G/–		NSP3	W147‡	
5291	T/C	4.21	C/–		NSP3	A429	
5546	G/–		G/A	4.87	Capsid		T514
7768	C/–		A/C	6.71	Capsid		A66V
7774	G/–		G/A	6.21	Capsid		R68H
8662	C/T	41.71	C/–		E2	A40V	
8728	C/T	6.38	T/–		E2	S62L	
8827	A/G	4.17	A/–		E2	H95R	
9195	A/G	4.29	A/–		E2	T218A	
9200	T/A	6.37	T/A	3.05	E2	D219E	D219E
9356	T/C	4.49	T/–		E2	P271	
10603	C/–		C/A	3.81	E1		T210N
11091	A/G	2.72	A/–		E1	S373G	
11303	A/–		T/C	4.82	3′ UTR		
11312	C/A	15.18	C/A	12.14	3′ UTR		
11450	T/–		T/C	6.90	3′ UTR		
11456	C/–		A/G	36.61	3′ UTR		

*Underlining indicates consensus nucleotide changes present in the serum compared with CSF sample. CSF, cerebrospinal fluid; NSP, nonstructural protein; SNV, single-nucleotide variants; UTR, untranslated region. Blank cells indicate not applicable in respective specimens. †Reference genome position based on isolate with GenBank accession no. KX029239. ‡Stop codon.

**Figure 2:**
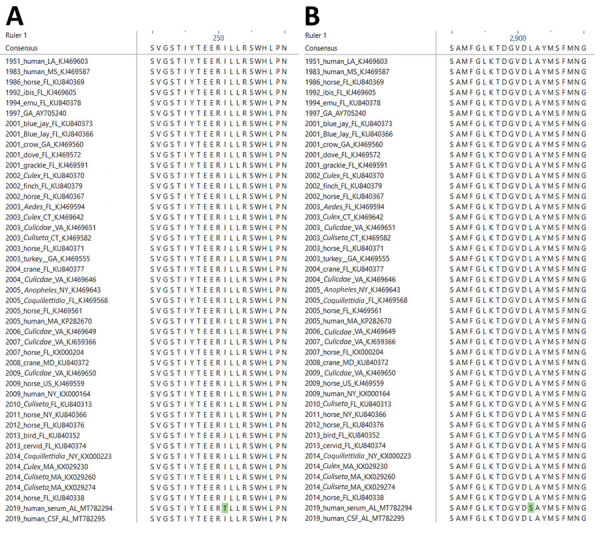
Amino acid sequence alignments depicting variation in the eastern equine encephalitis virus from a woman in Alabama, USA, 2019, compared with reference viruses. Open reading frames from 46 eastern equine encephalitis virus complete genomes were translated and aligned with ClustalW (*16*). Amino acid alignments show variants in the 2019 sequence in the nonstructural protein I251T (A) and structural E2 protein L62S (B). Green shading indicates changes unique to the virus sequence obtained from serum compared with cerebrospinal fluid. Reference viruses are labeled with year of isolation, host, state of isolation, and GenBank accession number.

Three consensus nucleotides found in the serum were not found in the CSF; however, the corresponding minor SNV populations at positions 775 (NSP1), 5291 (NSP3), and 8728 (E2) in the serum were detected in the CSF with 100% frequency. These consensus level viral populations in the serum resulted in 1 synonymous nucleotide substitution at nt 5921 in NSP3 and 2 nonsynonymous changes at 775 in NSP1-I251T and 8728 in E2-L62S compared with sequences from the CSF and reference EEEV isolates (Figure 2, panels A, B). These data suggest intrahost variability on minor viral populations as well as intrahost variability at the consensus level between the specimen sources.

## Discussion

EEEV causes a severe meningoencephalitis in equids, domestic birds, and humans. In 2019, the number of reported cases in humans increased substantially; 38 cases were confirmed in contrast with the annual average of 8. We confirmed EEEV infection in an immunocompromised person; deep sequencing of the viral RNA directly from the patient’s serum and CSF showed genetic relatedness to recent EEEV isolates in northern Florida and uniquely demonstrated EEEV intrahost variability in a human.

Very few sequences of EEEV isolates from Alabama have been described. The sequences from our study cluster within the FL4 (*12*,*17*) monophyletic clade with EEEV isolates from northern Florida collected during 2010–2014. These data support findings of a previous study that evaluated partial coding sequences of 3 isolates from mosquitoes in Alabama that suggest EEEV gene flow between Alabama and Florida (*18*). Of note, the EEEV sequences derived in our study did not phylogenetically associate with those from similar geographic areas in the Florida panhandle, which have been shown to have a unique spatial structure (*17*). This finding suggests a potentially complex ecologic association unrelated to geographic proximity. Future surveillance of EEEV in the region will help clarify whether similar FL4 clade strains continue to circulate or become extinct, as has often been observed in northern states (*12*).

Advances in sequencing have improved our knowledge of intrahost virus variation, or quasispecies, in several arboviruses, including WNV (*19*,*20*), dengue viruses (*21*), Venezuelan equine encephalitis virus (*22*,*23*), and Ross River virus (*24*); however, few studies have evaluated intrahost genetic variation for EEEV (*25*). Sequencing reads from the serum sample exhibited more viral variation, and sequencing reads from the CSF specimen identified fewer SNVs, especially in coding regions. Our data suggest that EEEV might face a genetic bottleneck between the blood and central nervous system because the genetic variability in the CSF was more limited. The reduction in genetic variability in the CNS could potentially result from a genetic bottleneck and subsequent founder effect because of transmission across the blood–brain barrier as has been observed with poliovirus (*26*). Alternatively, the genetic variability could be indicative of continued selection for viruses capable of replication in neuronal cells, possibly resulting in neurovirulence (*27*).

In addition to intrahost quasispecies diversity, we also observed variation in the consensus sequences derived from each specimen. The consensus sequence derived from serum had 2 nonsynonymous nucleotide changes compared with that of the CSF. One amino acid change, NSP1-I251T, is located in an amphipathic peptide that has been shown to play a role in the membrane association of NSP1 (*28*), possible cell-to-cell transmission, and pathogenicity of alphaviruses (*29*,*30*). The second change, E2-L62S, is within the A domain in the wing region (*31*). This domain has been implicated in neutralization epitopes for several alphaviruses (*32*–*35*) and has also been demonstrated to be involved with heparin sulfate receptor binding in neuronal cells (*36*,*37*).

When evaluating both intrahost virus variants and consensus-level majority variation, we found decreased variation in the CNS is not altogether unexpected because of potential bottlenecks and selection. It is noteworthy that consensus level amino acid changes observed in the serum are not reflected in the CNS. Stochastic generation of virus variants and lack of immune selection cannot explain fixation of 2 nonsynonymous amino acid changes in the peripheral compartment. It is possible that this scenario fits the quasispecies model of cooperative interaction in the virus population as described for poliovirus (*38*). Applying our observations to the quasispecies model (*39*) leads to the suggestion that the virus diversity in the periphery could contribute to systematic spread by maintaining the viral subpopulations that might facilitate CNS invasion and replication in this unique compartment. Although this study and observation are limited by a single description of EEEV in human serum, future surveillance and sequencing will add to our knowledge of EEEV disease and virus diversity.

The virus sequences generated in this study were derived from serum and CSF specimens from an immunocompromised person with no detectable serologic antibody response to EEEV, probably because of rituximab therapy for lymphoma. Patients receiving B cell–depleting monoclonal antibody therapy may be predisposed to severe neuroinvasive disease and death after arbovirus infection. Cases have been associated with prolonged RNA detection in serum and CSF or brain tissue and lack of serologic response (*40*,*41*). This unique circumstance enabled us to sequence EEEV directly from the serum and CSF without amplification and report the complete EEEV sequence derived from human serum. The patient’s Cq values of EEEV in serum were low, and viral genome diversity was broad. Although the relative Cq values observed in this study are similar to those found in *Cs. melanura* mosquitoes with high EEEV titers (*42*), they are below virus titers that have been observed in experimentally infected birds (*43*). It is unknown if the viral load in immunocompromised persons could lead to subsequent acquisition and transmission of the virus by a mosquito, but we can speculate that these persons could be hosts for mosquitoborne viruses, given higher viral loads and more prolonged viremias than these observed in dead-end hosts (*44*–*46*). 
